# Towards targeted combinatorial therapy design for the treatment of castration-resistant prostate cancer

**DOI:** 10.1186/s12859-017-1522-2

**Published:** 2017-03-22

**Authors:** Osama Ali Arshad, Aniruddha Datta

**Affiliations:** 1 Department of Electrical and Computer Engineering, Texas A&M University, College Station, TX USA; 2 Center for Bioinformatics and Genomics Systems Engineering, Texas A&M University, College Station, TX USA

**Keywords:** Prostate cancer, Gene regulatory networks, Boolean modeling, Combination therapy, Stochastic logic, Vulnerability assessment

## Abstract

**Background:**

Prostate cancer is one of the most prevalent cancers in males in the United States and amongst the leading causes of cancer related deaths. A particularly virulent form of this disease is castration-resistant prostate cancer (CRPC), where patients no longer respond to medical or surgical castration. CRPC is a complex, multifaceted and heterogeneous malady with limited standard treatment options.

**Results:**

The growth and progression of prostate cancer is a complicated process that involves multiple pathways. The signaling network comprising the integral constituents of the signature pathways involved in the development and progression of prostate cancer is modeled as a combinatorial circuit. The failures in the gene regulatory network that lead to cancer are abstracted as faults in the equivalent circuit and the Boolean circuit model is then used to design therapies tailored to counteract the effect of each molecular abnormality and to propose potentially efficacious combinatorial therapy regimens. Furthermore, stochastic computational modeling is utilized to identify potentially vulnerable components in the network that may serve as viable candidates for drug development.

**Conclusion:**

The results presented herein can aid in the design of scientifically well-grounded targeted therapies that can be employed for the treatment of prostate cancer patients.

## Background

Prostate cancer is the most common noncutaneous male malignancy and one of the leading causes of cancer mortality in the western world [1]. The growth and progression of prostate cancer is stimulated by androgens [2]. Androgens are male sex steroid hormones that are responsible for the development of male characteristics. Testosterone is the most important androgen in men. The effects of androgens are mediated through the androgen receptor (AR) [3]. The androgen receptor is a nuclear receptor, which is activated in response to the binding of androgens. Upon activation, it mediates transcription of target genes that modulate growth and differentiation of prostate epithelial cells. In malignant prostate cells, androgen signaling is deregulated and the homeostatic balance between the rate of cell proliferation and programmed cell death is lost. As prostate cancer relies on androgens for growth, the main line of treatment focuses on abrogating the action of androgens. Androgen deprivation therapy (ADT) in the form of surgical or medical castration is the cornerstone of treatment for prostate cancer [4]. Initially, androgen ablation induces significant regression of the tumor. However, the response to ADT is temporary and prostate cancer invariably stops responding to this treatment regimen, leading to a clinical condition that is known as hormone-refractory prostate cancer, androgen-independent prostate cancer or castration-resistant prostate cancer (CRPC). CRPC is a more aggressive and typically lethal phenotype where the tumor continues to grow in spite of the very low levels (<50 ng/ml) of circulating serum testosterone. Standard treatment options are limited and palliative docetaxel-based chemotherapy is generally used for patients who have become refractory to hormone treatment. However, median survival time for patients following first-line chemotherapeutic treatment is just eighteen to twenty-four months [5]. There is therefore a clear rationale for advances in alternative therapeutics in order to evolve and expand the landscape of treatment options for malignant forms of prostate cancer that recur after abatement.

Over recent years, there has been a significant effort towards furthering our understanding of the molecular mechanisms underpinning tumor development, growth and progression. It is now appreciated that in spite of castrate levels of androgens, the cancer cells are able to maintain persistent androgen receptor signaling through a variety of contributory mechanisms including AR gene amplification that results in overexpression of AR, gain-of-function mutations in AR which enable promiscuous activation of the receptor through other steroids or even in the absence of ligand binding, changes in AR co-activators and the expression of AR splice variants [6]. This compensatory response allows cancer cells to survive in a low testosterone environment and the reactivated AR signaling axis continues to play a role after neoplastic transformation. Additionally, certain androgen-independent cellular signaling pathways that promote proliferation and inhibit apoptosis, have been critically implicated as drivers of continued progression of prostate cancer. Hence, accumulating evidence indicates that the growth and progression of prostate cancer is a complicated process that involves interaction between multiple pathways. Advances in our knowledge of the biology of prostate cancer has led to the development of a number of novel therapies designed to target signaling pathways involved in disease progression. With the exception of certain androgen synthesis and AR signaling antagonists that have received regulatory approval, these advanced agents are under various stages of clinical trials [7].

Castration-resistant prostate cancer is a complex malady. Given the inherent complexity of the CRPC signaling cascade, there is no one dominant molecular driver across all tumors and hence no single drug can act as a “magic bullet” by being uniformly effective for treating the malignancy [8, 9]. At best, limited benefit will be derived from targeting a single molecule. Rational combinations of signal-modulating therapeutic agents have higher likelihood of yielding better outcomes. While there are several drugs being tested on cell lines, most of these studies focus on a single pharmaceutical agent and very few of those experiments involve trying out drug combinations. Furthermore, prostate cancer is a markedly heterogeneous disease, with different tumors varying in their composition and makeup. In other words, different tumors will harbor different malfunctions in the signaling pathways. Thus, tailored targeted therapies based on individual tumor characteristics are required to maximize the potential benefits from treatment.

Mathematical and computational modeling plays a pivotal role in systems biology in elucidating biological insights from large-scale biomolecular signaling networks that are not amenable to straightforward intuitive interpretation. A diverse array of formalisms have been proposed in this domain as suitable representations for complex multicomponent networks such as cellular signaling pathways [10]. Amongst these frameworks, Boolean network models [11, 12] have emerged as an extremely useful parameter-free approach to capture the qualitative behavior of extensive genetic networks wherein knowledge of kinetic parameters is scarce. Boolean logic models have been successfully applied to study biological signaling networks and cellular processes [13, 14], for instance the cell cycle [15], apoptosis [16], the T cell survival network [17], hypoxia stress response pathways [18] and the gene regulatory network regulating cortical area development [19]. In this paper, we use Boolean logic modeling of the key signaling pathways implicated in the development and progression of prostate cancer to simultaneously test various combinations of agents for their efficacy in attenuating cancer growth and design targeted therapies for the management of the disease. In addition, we attempt to delineate components in the signaling network that can be pharmacologically manipulated to therapeutic advantage.

## Methods

### Prostate cancer signal transduction network

**Fig. 1:**
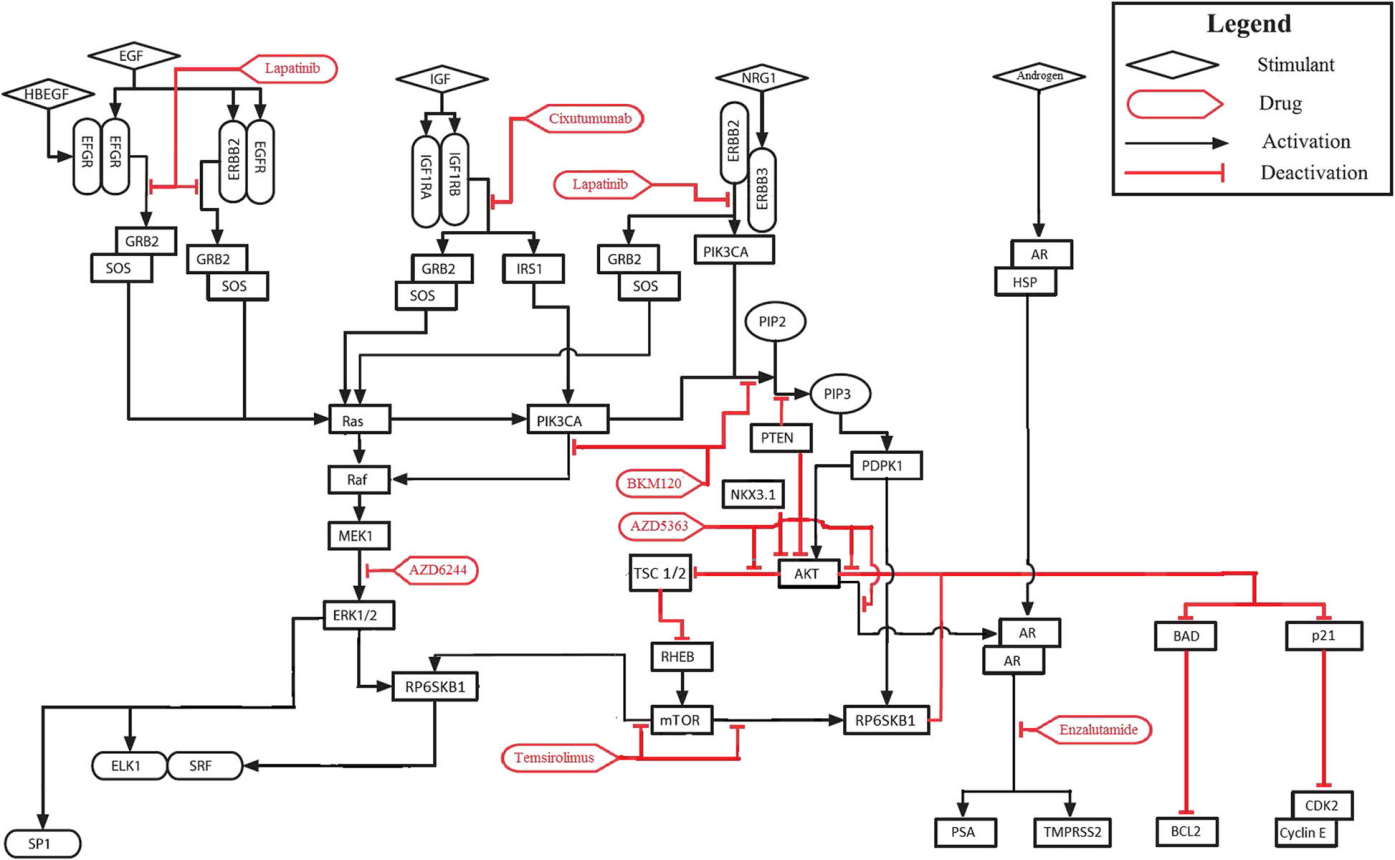
Prostate cancer signal transduction network. A schematic diagram of key signaling pathways deregulated in prostate cancer. *Black* and *red lines* represent activating and inhibiting interactions respectively whereas the *red boxes* depict prostate cancer drugs at their corresponding points of intervention in the network

Cellular processes such as growth and division are regulated by an interconnected network of molecules referred to as signaling pathways. Key cellular signal transduction pathways known to play a major role in cell survival, growth, differentiation and the development of castration-resistance in prostate cancer are the Androgen Receptor (AR), PI3K/AKT/mTOR and Mitogen-Activated Protein Kinase (MAPK) pathways. The aberrant behavior of prostate cancer cells is characterized by dysfunction in these selective oncogenic signaling pathways promoting malignant characteristics. These pathways play a role in a diverse range of essential physiological cellular processes such as differentiation, survival, proliferation, protein synthesis and metabolism. Malfunctions in these pathways are common in prostate cancer malignancies. For example, approximately 70% of advanced prostate cancers have genomic alterations in the PI3K/AKT/mTOR pathway [20]. These three pathways are the most frequently over-activated pathways increasing survival of cancer cells and promoting cancer progression [21]. A schematic representation of these pathways is shown in Fig. 1 [22–24]. The pharmacologic agents depicted in red boxes in the figure are highly specific pathway inhibitors. These reagents modulate growth-factor receptors and the downstream pathways abnormally activated in CRPC by targeting with great specificity certain signaling nodes in the network.

### Boolean modeling of prostate cancer signaling

**Fig. 2:**
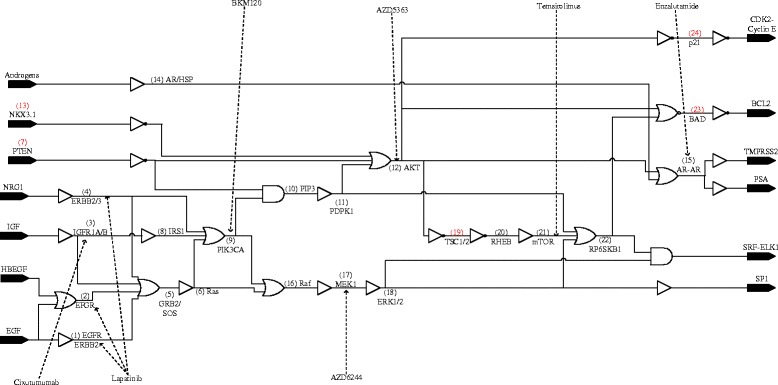
Boolean model. Combinational circuit model of prostate cancer signaling pathways. Each node is assigned a numeric label in parentheses. These labels also serve to enumerate the fault locations with stuck-at-one and stuck-at-zero faults in black and red numerals respectively. The dotted arrows indicate the intervention points for the respective drugs

In the context of methodologies that are applied to model cellular signal transduction networks, Boolean networks are probably the simplest where the state of each node in the network is either active (on) or inactive (off). In a Boolean network, the nodes are the genes and the edges represent the interaction amongst the genes. Since the molecules in a gene-regulatory-network (GRN) exhibit switch-like behavior, genes may be regarded as binary devices where a gene can be considered to be active if it is being transcribed and inactive if it is not. Moreover, the relationships amongst the genes may be represented by means of logical functions. Thus, a GRN is amenable to such a representation. The Boolean formalism is analogous to a digital circuit where logic gates can be used to represent the regulatory relationships amongst the nodes and the activation level of the nodes is indicated by binary logic. The biological interactions amongst the various nodes (genes) represented in the gene regulatory network of Fig. 1 can therefore be translated to an equivalent Boolean circuit [25]. Let’s say either gene X or Y can activate a third gene Z, then we can model this component of the signaling network with an OR gate with two inputs, namely X and Y and with output Z. Thus, the signaling network of Fig. 1 can be mapped to the combinational circuit shown in Fig. 2. This digital logic circuit represents our multi-input multi-output (MIMO) systems model of the prostate cancer signaling transduction network.

**Fig. 3:**
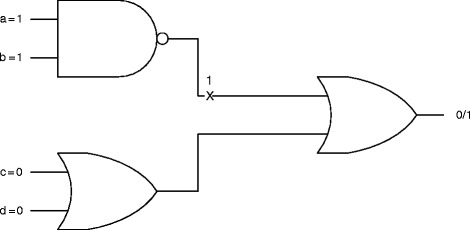
Circuit with stuck-at fault. An example of a stuck-at fault. In the absence of the stuck-at fault, the output is zero. If there is a stuck-at-one fault at the location marked with a cross, the output of the faulty circuit becomes one

Cancer is a disease of abnormal cell signaling caused by a breakdown in the normal signaling pathways leading to the loss of cell cycle control and uncontrolled cell proliferation. These abnormalities in the signaling network can be represented as stuck-at faults [26]. A stuck-at fault is said to occur when a line in the network is permanently set to a fixed value of one (stuck-at-one fault) or zero (stuck-at-zero fault) with the result that the state of the line is stuck at the faulty value and no longer depends on the state of the signaling network upstream that drives that line i.e. the faulty line has a constant (1/0) value independent of other signal values in the circuit. A stuck-at-fault can occur either at the input or output of a gate. An example of a stuck-at-fault is given in Fig. 3. Suppose the input vector is <abcd>= 1100. In this case, the output is 0. However, if there is a stuck-at-one fault at the output of the NAND gate with the same input vector as before, the output of the faulty circuit is one instead of zero. This notion of stuck-at-faults has immediate biological relevance: on account of mutations or other structural abnormalities, a gene might become dysfunctional and hence stuck at a particular state irrespective of the signals that it is receiving from surrounding genes [27]. These biological defects can be abstracted as stuck-at faults. For instance, as discussed earlier, a diverse array of mechanisms engender persistent AR signaling in CRPC even with castrate serum levels of androgen. This constitutive (permanent) activation of the androgen receptor where the receptor remains active i.e. continues to signal downstream even in the absence of androgens can be represented as a stuck-at-1 fault. By the same token, the inactivation in cancer of a tumor suppressor, which acts as a molecular brake on cell growth in a normal cell, can be represented as a stuck-at-0 fault. From our Boolean circuit model, we can explicitly enumerate the different locations where a fault can occur. These fault locations are numbered in Fig. 2 with the stuck-at-0 and stuck-at-1 faults in red and black numerals respectively. There is a total number of 24 possible fault locations.

The objective is to counteract the effect of these faults by targeted drug intervention, so we incorporate the drugs in our model. The drug intervention points are illustrated in Fig. 2 which are the locations of the molecules that these prostate cancer drugs are known to target. Since the drugs inhibit the activity of their target i.e. the main mechanism of action of the anti-cancer drugs is to cut off downstream signaling, their action is incorporated in our model as an inverted input to an AND gate with the result that whenever the drug is applied, the gene that it targets is turned off.

### Simulation for fault mitigation with drug intervention

We can now use our Boolean model to test different combination therapies in terms of their efficacy in mitigating the effects of the faults. For each fault, we would like to intervene with the best possible drug combination i.e. we want to determine which set of drugs would be most effective in attempting to nullify the effect of that fault, thereby providing us with a targeted therapy based on the tumor signature. Define, the input vector as follows:

$$\begin{aligned} {}INPUT = \left[\operatorname{EGF}, \operatorname{HBEGF}, \operatorname{IGF}, \operatorname{NRG1}, \operatorname{PTEN}, \operatorname{NKX3.1}, \operatorname{Androgens}\right] \end{aligned} $$

The first four components of this vector are growth factors, which are external signals that stimulate a cell to grow and replicate. The next two input components, namely PTEN and NKX3.1 are tumor suppressors which act as molecular brakes on cell division. The last input vector component consists of the external hormones that stimulate the AR pathway in a normal prostate cell. The input vector is set to be [0000110]. This corresponds to all the external signals that stimulate cell growth being absent and the molecular brakes being active i.e. this input vector corresponds to a non-proliferative input which produces a non-proliferative output in the fault-free case. The output vector is defined to be:

$$\begin{aligned} {}OUTPUT = \left[\operatorname{SP1}, \operatorname{SRF-ELK1}, \operatorname{PSA}, \operatorname{TMPRSS2}, \operatorname{BCL2}, \operatorname{CDK2-Cyclin E}\right] \end{aligned} $$

The output vector consists of key markers of cell growth and proliferation in prostate cancer. In the fault-free scenario, a non-proliferative input to the regulatory network should produce a non-proliferative output characterized by the all-zero vector. However, faults in the network will produce a non-zero (proliferative) output even when the input is non-proliferative. The objective is to drive the faulty network’s output as close as possible to that of the fault-free circuit i.e. towards the all-zero vector through targeted drug intervention. Define, the drug vector as:

$$\begin{aligned} {} DRUG \ VECTOR = \left[\operatorname{Lapatinib}, \operatorname{Cixutumumab}, \operatorname{AZD6244}, \operatorname{BKM120}, \operatorname{AZD5363},\right.\\ \left.\operatorname{Temsirolimus}, \operatorname{Enzalutamide}{\vphantom{\operatorname{Lapatinib}}}\right] \end{aligned} $$

Each component of the drug vector is one if the corresponding drug is applied and is zero otherwise i.e. the i^th^ bit of the drug vector is one if the drug is selected and zero if it is not. Thus, for example, the drug vector [0010010] represents the combination of AZD6244 and Temsirolimus. Since, the total number of drugs is seven, the number of possible drug combinations is 128. The objective is to determine the best possible therapy for each fault. Each fault represents a different molecular abnormality and hence a tumor with a different profile.

For each of the faults, the problem is to find the drug selection that can rectify the fault i.e. change the faulty output to the correct output. If that is not possible, the best drug vector will drive the output as close as possible to the fault-free output. A simple metric that can be used as a distance measure to determine how far the output vector is from the fault-free vector is Hamming distance. Faults that produce an output vector with a greater Hamming distance from the correct output have more of the proliferative genes active and presumably a greater proliferative effect. Since the correct output is the all-zero vector, the Hamming distance of the output vector from the correct output is simply the Hamming weight of the output vector (for binary vectors Hamming weight is equivalent to the *L*_1_-norm). For each fault, we determine the output under every possible drug vector. The best therapy for that fault is the drug vector that produces the output with the smallest Hamming weight. In addition, since the drugs have deleterious side-effects, we would like to choose a drug combination with the fewest number of drugs. Thus, the best targeted therapy for each of the cancer-inducing faults is the one that under the presence of the fault, produces the best output with the smallest Hamming weight with the minimal number of drugs.

To determine the best combination therapy across all faults, for each drug combination we determine the sum of the Hamming weights of the output vector across all possible combinations of faults and choose the drug combination that yields the smallest total. In order to keep the computation tractable, we restrict the number of possible faults in any fault combination to be no more than three i.e. up to three genes can be faulty simultaneously. We constrain the cardinality of the drug vector to be less than or equal to three, in essence limiting the number of drugs in the combination to three since on account of the harmful side-effects of the drugs, administering four or more cancer drugs simultaneously might not be prudent.

Let us formalize the qualitative description above of the selection of best therapy for each fault and that of the overall optimal drug vector. For the Boolean network (BN) of Fig. 2, let *N*,*M* and *P* be the total number of primary inputs, primary outputs and fault locations respectively, then *N*=7, *M*=6 and *P*=24. Let $\mathbf {x} \in \mathcal {X}$ and $\mathbf {z} \in \mathcal {Z}$ be the input and output vectors respectively where $\mathcal {X}$ and $\mathcal {Z}$ represent the space of all binary vectors of dimensions *N* and *M* respectively. Let *x* ^∗^=[ 0,0,0,0,1,1,0] be the input vector corresponding to the non-proliferative input.

Let *D* represent the total number of drug combinations (vectors) with no more than three drugs in any combination, then $D=\sum \limits _{k=0}^{3}\binom {7}{k}$. Denote each drug vector in the drug space as *d*_*i*_with *i*=0,…,*D*−1 (*d*_0_ is the all-zero drug vector meaning no drug is applied). Let $\mathcal {D}$ be this space of drug vectors.

Let *C* be the total number of fault combinations with no more than three faults in any combination, then $C=\sum \limits _{k=0}^{3}\binom {P}{k}$. Assign each fault combination in the fault space a label *f*_*j*_ with *j*=0,…,*C*−1 (*f*_0_ represents the fault-free case). Let $\mathcal {F}$ be this set of faults.

Let ***ψ*** denote the mapping from a given input vector, drug combination and fault combination to an output vector: $\mathbf {x} \in \mathcal {X}, \mathbf {d} \in \mathcal {D}, f \in \mathcal {F} \xrightarrow {\boldsymbol {\psi }} \mathbf {z} \in \mathcal {Z}$ i.e. ***ψ*** represents the output of the BN for a given input **x** when a drug combination **d** is applied under fault scenario *f*. Let *ψ*_*i*_ be the i^th^ component of this M-dimensional vector ***ψ***.

The best drug vector *d*_*i*_, *i*∈{0,1,…,*D*−1} for each single fault *f*_*j*_, *j*∈{1,2,…,*P*} is the vector of smallest Hamming weight that minimizes ∥***ψ***(*x* ^∗^,*d*_*i*_,*f*_*j*_)∥_1_.

The optimal drug combination across all faults is:

1 $$ \begin{aligned} \mathbf{d_{i}^{*}} = {\underset{\mathbf{d_{i}}}{\arg\min}} \sum\limits_{j=1}^{C-1} \left\|{\boldsymbol{\psi}\left(\mathbf{x^{*}}, \mathbf{d_{i}}, f_{j}\right)}\right\|_{1} \end{aligned}  $$

$\mathbf {d_{i}^{*}}$ is determined by exhaustive enumeration by explicitly searching for the drug combination that for a non-proliferative input, minimizes the sum of Hamming weights (*L*_1_-norms) of the output vector across all possible combinations of faults.

### Node vulnerability assessment

In electronic circuits, reliability refers to the probability of a circuit functioning as intended i.e. producing the correct output. Reliability assessment is used to determine the vulnerability of a circuit to faults. A number of different techniques have been proposed for reliability analysis in digital circuits [28]. Recently, in [29] a scalable, efficient and accurate simulation-based framework based on stochastic computations was introduced for logic circuit reliability evaluation. In biological systems, dysfunctions in nodes in the signaling network cause deviation from normative behavior. Reliability assessment methodologies can be leveraged on Boolean network models of pathways to determine the vulnerability of the network to the dysfunction of each node [30, 31]. In this section we conduct a stochastic logic based vulnerability analysis of the prostate cancer signal transduction network in order to discover the most vulnerable nodes thereby allowing us to prioritize such segments in the network whose perturbation has the greatest potential to yield the most clinical benefit.

In stochastic logic, signal probabilities are encoded in random binary bit streams (the signal probability of a node corresponds to the likelihood of that node having logic value one). For example, the binary sequence 0110010100 of length ten encodes the probability 0.4 since the proportion of ones in this sequence is $\textstyle \frac {4}{10}$. In practice, the length of the stochastic sequences typically used is much larger. Since the biological literature is devoid of precise ligand binding probabilities, each primary input is assumed equally likely to be 0 or 1 i.e. all primary input signal probabilities are taken to be 0.5.

Stochastic logic often makes use of Bernoulli sequences for the random binary streams where each bit in the stream is generated independently from a Bernoulli random variable with a probability of one equal to *p*. The use of probabilistic sequences inevitably introduces stochastic fluctuations which implies that the result produced is non-deterministic. These fluctuations can be significantly reduced by representing the initial input probabilities by non-Bernoulli sequences [32] defined as random permutations of sequences containing a fixed number of ones and zeros. For a given probability *p* and sequence length *L*, a non-Bernoulli sequence contains a fixed number *pL* of ones, with the positions of the ones determined by a random permutation. Thus, for example, to represent the probability 0.5 by a non-Bernoulli stream of length 10, we could randomly permute the sequence 1111100000 which has five ones (instead of generating each bit from a Bernoulli random variable with *p*=0.5 as would have been done to represent the same probability by a Bernoulli sequence). We use non-Bernoulli sequences of random permutations of fixed number of ones and zeros in order to encode the initial input probabilities.

**Fig. 4:**
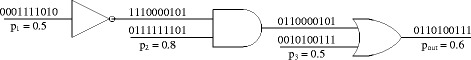
A stochastic logic circuit. An example of a stochastic logic circuit

A logic circuit operating on stochastic bit streams (see Fig. 4 for an example), accepts as input random sequences representing the probability of each input being one and produces ones and zeros like any digital circuit [33] i.e. a stochastic logic circuit uses Boolean gates to operate on sequences of random bits. Each bit-stream represents a stochastic number interpreted as the probability of seeing a one in an arbitrary position. Thus, the computations performed by such a circuit are probabilistic in nature. The output bit stream produced can be decoded as the probability of the output being one by counting the number of ones in the stream and dividing by its length.

**Fig. 5:**
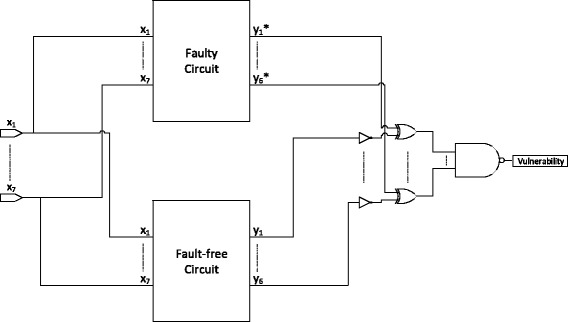
Computation of node vulnerability. Depicts the architecture used to compute the vulnerability of a node. *x*_1_ to *x*_7_ are the input stochastic bit streams for each of the seven primary inputs in the Boolean network model. The output bit streams for each of the six output components when these input sequences are propagated through the circuit with a dysfunctional node (whose vulnerability we want to compute) are denoted by $y_{1}^{*}$ to $y_{6}^{*}$ whereas those for the fault-free circuit are labeled as *y*_1_ to *y*_6_

The vulnerability of a node is defined as the probability that the system produces incorrect output if that particular node is dysfunctional (faulty) i.e. it is the probability that the output of the network is different when that node is dysfunctional and is the complement of reliability. The procedure to determine the node vulnerabilities is illustrated in Fig. 5 is as follows. We generate non-Bernoulli sequences of length L=1,000,000 in which exactly half of the bits are set to one at each of the seven initial inputs. The input stochastic sequences are propagated through both the original error-free circuit and the circuit in which the node of interest is dysfunctional. As discussed in the previous section, the dysfunction of a node is represented by a corresponding stuck-at fault of the requisite type at the particular location. This produces two sets of stochastic bit streams, one at each of the primary outputs of the fault-free circuit and the other at the primary outputs of the unreliable circuit. The proportion of ones in the output bit stream encodes the output signal probabilities i.e. the probability of the output being one. Since the reliability of the circuit under the fault is the probability that the circuit output is same as that of the fault-free circuit, the sequence encoding the output reliability can be obtained from the output sequence of the faulty circuit by comparing it to the output sequence of the fault-free circuit and setting each bit to one whenever the corresponding bits in the sequences are the same and zero if they are different. The proportion of ones in this resulting sequence will then correspond to the reliability of that output. Thus, we can obtain the stochastic sequence representing the reliability of each output by taking the XOR of each output bit stream of the faulty circuit with the complement of the corresponding output bitstreams of the fault-free circuit. For a circuit with multiple primary outputs as is the case here, the stochastic sequence encoding the joint output reliability can be obtained by taking the stochastic AND of the outputs of the XOR gates as the stochastic AND operation on the output of XOR gates produces a one only if all the corresponding bits at each XOR gate are one i.e. if all the corresponding bits in the respective outputs of the fault-free and faulty circuit are same. We then take the complement of the bit stream at the output of this AND gate to obtain the stream that encodes vulnerability. This bit stream can then be decoded to determine the node vulnerability with the proportion of ones in this stream equivalent to the vulnerability of the node.

The procedure for computing the vulnerability of a node described above and depicted in Fig. 5 is summarized as follows:

- Generate non-Bernoulli streams encoding input probabilities at each of the primary inputs.
- Propagate the input binary streams through the fault-free circuit and obtain a random bit sequence for each output.
- Propagate the same input binary streams through the circuit with a stuck-at fault at the location of the node whose vulnerability we want to determine and again obtain a random bit sequence for each output.
- XOR each primary output sequence from the faulty circuit obtained in step 3 with the complement of the corresponding primary output sequence from the fault-free circuit.
- AND all the sequences obtained from each XOR gate. Take the complement of the stream so obtained. The vulnerability of the node is the fraction of ones in the resulting bit stream.

Thus, in a nutshell, the node vulnerabilities are obtained by propagating the initial input stochastic bit streams encoding the input probabilities through both the faulty and fault-free circuit, comparing the respective outputs obtained from each and decoding probabilities from the resulting streams.

Let *x*_1_,*x*_2_,…,*x*_*N*_ represent input non-Bernoulli sequences of length *L* with each sequence represented as a vector of length *L* whose i^th^ component is equal to the i^th^ bit in the sequence. Define the *L*×*N* matrix $X=(\mathbf {x}_{1}^{\top }\;\mathbf {x}_{2}^{\top }\;\dotsc \;\mathbf {x}_{N}^{\top })$. Thus, each row of this matrix contains the corresponding bits of each of the primary input streams. The vulnerability *v*_*j*_ of node *j*∈{1,2,…,*P*} is given by:

2 $$ \begin{aligned} v_{j} = \frac{1}{L} \sum\limits_{k=1}^{L} \left(\prod\limits_{i=1}^{M} \psi_{i}\left(\mathbf{x}=\left[X_{k1}, \dotsc, X_{kN}\right], \mathbf{d_{0}}, f_{j}\right)\right. \\\oplus \left. \psi_{i}^{\prime}\left(\mathbf{x}=\left[X_{k1}, \dotsc, X_{kN}\right], \mathbf{d_{0}}, f_{0}\right) {\vphantom{\prod\limits_{i=1}^{M}}}\right)^{\prime} \end{aligned}  $$

where ^′^ is the bit-complement operator and ⊕ is the binary XOR operator.

## Results and discussion

### Simulation results for drug intervention

We use the Boolean network model to determine an apposite therapy for each fault. As described in the methods section, the best targeted therapy for each of the cancer-inducing faults is the one that under the presence of the fault, produces the output with the smallest Hamming weight with the minimal number of drugs. The best therapy for each of the faults is shown in table 1 with the drug vector defined as before. Note that for certain faults, no drug vector can improve the output. Such faults are said to be untestable since no test (drug vector in this case) can rectify the fault. This is because there are no drugs on the fan-out of these genes. However, all these faults with the exception of fault 18 are minimally proliferative as they produce a faulty output with the least possible Hamming weight of one.

**Table 1:** Best therapy for each fault

Fault location	Drug vector
1	1000000
2	1000000
3	0100000
4	1000000
5	0011000
6	0011000
7	0000100
8	0001000
9	0001000
10	0000100
11	0000100
12	0000100
13	0000100
14	0000001
15	0000001
16	0010000
17	0010000
18	0000000
19	0000010
20	0000010
21	0000010
22	0000000
23	0000000
24	0000000

Thus, there are many locations in the gene regulatory network of prostate cancer where malfunctions can occur resulting in a cancer that is different, requiring a specific targeted therapy. The table facilitates arriving at such a therapy as it maps each malfunction to an appropriate set of drugs. The look-up table can be used to devise therapies that have a higher likelihood of success since they are tailored specifically to the molecular abnormalities in critical pathways and thereby facilitates an individualized approach to therapy design.

In order to find the best combination therapy across all possible faults, as discussed in the methods section, for each drug combination we determine the sum of the Hamming weights of the output vector across all possible combinations of faults and choose the drug combination that yields the smallest total. This gives us the drug cocktail of AZD6244, AZD5363 and Enzalutamide as a combination therapy for advanced prostate cancer. In a recent study, the drug combination of AZD5363 and Enzalutamide has demonstrated an impressive response in prostate cancer models [34]. Moreover, AZD6244 in partnership with an AKT pathway inhibitor (analogous to AZD5363), has been proposed as a strategy for the treatment of CRPC [35]. Thus, we propose that the aforementioned drug triad which represents a horizontal blockade approach, wherein combination therapy is used for the concerted pharmacologic inhibition of multiple compensatory pathways, as a therapeutic modality that may attenuate prostate cancer survival and growth.

### Node vulnerabilities

Using the framework delineated in the methods section, we quantify the vulnerability of different nodes. The vulnerability values obtained are given in Table 2. Vulnerability assessment can be used to identify candidates for targeted drug development. Nodes whose vulnerabilities are higher should be presumably better targets for drugs since potentially therapeutic benefit is more likely for nodes which are more vulnerable. We observe that the AR-mediated signaling axis remains a valid target. Furthermore, we see that dysfunction in the AKT nexus and the loss of tumor-suppressors have higher vulnerability values so drugs that attempt to alleviate these aberrations should be efficacious in attenuating tumor growth. The design of anti-cancer therapeutics directed at the loss of tumor suppressors has been difficult [36]. Additionally, AKT-selective drug development is challenging due to its homology with other kinases [37]. These complications notwithstanding, accelerated development of novel agents that target these aberrations is warranted. In contrast, the vulnerabilities for certain nodes such as those in the mTOR axis are low indicating that they might not be attractive targets for drug development. Indeed, marginal clinical activity has been observed for mTOR inhibition with agents such as everolimus and temsirolimus failing to impact tumor proliferation in men with prostate cancer [4, 38]. Finally, in terms of the key pathways implicated in the disease we see that castration-resistant prostate cancer shows most vulnerability on aggregate to dysfunction in the AKT pathway. In a study it was demonstrated that the AKT pathway dominates AR signaling in CRPC [39].

**Table 2:** Node vulnerabilities

Node	Vulnerability (%)
1	6.25
2	6.25
3	6.25
4	6.25
5	6.25
6	6.25
7	24.98
8	6.25
9	6.25
10	24.98
11	24.98
12	24.98
13	24.98
14	12.47
15	12.47
16	6.25
17	6.25
18	6.25
19	1.57
20	1.57
21	1.57
22	1.57
23	1.57
24	24.98

## Conclusion

Castration-resistant prostate cancer is a hormone refractory phenotype of significant morbidity and mortality in the prostate cancer disease continuum where patients no longer respond to androgen ablation therapy. The biomolecular network representing the signaling pathways involved in the pathogenesis of this lethal malignancy is translated to a digital circuit. The locations of possible malfunctions in the digital circuit are identified and computer simulation of the equivalent model is used to predict effective therapies that mitigate the effect of different faults. A prospectively attractive combinatorial therapeutic strategy for the constellation of abnormalities is to leverage an AR axis targeted agent in conjunction with reciprocal inhibitors of other dysregulated pathways that are fundamental in coordinately driving oncogenesis. Proof of principle of clinical use for the proposed regimen remains to be demonstrated. A reliability (vulnerability) analysis methodology of digital circuits premised on stochastic logic modeling is utilized to quantify the vulnerability of the network to the dysfunction in discrete components in the signaling cascade thereby identifying key variables as targets for intervention that conceivably might be exploited by a new generation of novel therapeutics. These findings can contribute to the development of new rational approaches for the possible treatment of androgen-refractory prostate cancer. There is however a paucity of companion predictive biomarkers that can be used for the stratification of patients based on molecular aberrations in order to prescribe the apposite treatment. Furthermore, the histological and clinical heterogeneity of CRPC and the inherent redundancy along with the presence of feedback loops in pathways whose molecular underpinnings in the context of the disease induction and development are not yet fully understood, tender any potential translation into objective clinical efficacy of therapeutic implications derived from computations fraught with challenges.

## Abbreviations

ADT: Androgen deprivation therapy
AR: Androgen receptor
BCL2: B-cell lymphoma 2
BN: Boolean network
CDK2: Cyclin dependent kinase 2
CRPC: Castration resistant prostate cancer
EGF: Epidermal growth factor
GRN: Gene regulatory network
HBEGF: Heparin binding EGF-like growth factor
IGF: Insulin-like growth factor
MAPK: Mitogen activated protein kinase
MIMO: Multi-input multi-output
NRG1: Neuregulin 1
PSA: Prostate specific antigen
PTEN: Phosphatase and tensin homolog
SP1: Specificity protein 1
TMPRSS2: Transmembrane protease serine 2
